# Comprehensive multiphase NMR applied to a living organism†
†Electronic supplementary information (ESI) available. See DOI: 10.1039/c6sc00329j


**DOI:** 10.1039/c6sc00329j

**Published:** 2016-04-18

**Authors:** Yalda Liaghati Mobarhan, Blythe Fortier-McGill, Ronald Soong, Werner E. Maas, Michael Fey, Martine Monette, Henry J. Stronks, Sebastian Schmidt, Hermann Heumann, Warren Norwood, André J. Simpson

**Affiliations:** a Department of Physical and Environmental Science , University of Toronto , 1265 Military Trail , Toronto , ON , Canada M1C 1A4 . Email: andre.simpson@utoronto.ca; b Bruker BioSpin Corp. , 15 Fortune Drive , Billerica , Massachusetts , USA 01821-3991; c Bruker BioSpin Canada , 555 Steeles Avenue East , Milton , ON , Canada L9T 1Y6; d Silantes GmbH , Gollierstr. 70 C , 80339 München , Germany; e Environment Canada , 867 Lakeshore Rd. , Burlington , ON , Canada L7R 4A6

## Abstract

Comprehensive multiphase (CMP) NMR is a novel technology that integrates all the hardware from solution-, gel- and solid-state into a single NMR probe, permitting all phases to be studied in intact samples. Here comprehensive multiphase (CMP) NMR is used to study all components in a living organism for the first time. This work describes 4 new scientific accomplishments summarized as: (1) CMP-NMR is applied to a living animal, (2) an effective method to deliver oxygen to the organisms is described which permits longer studies essential for in-depth NMR analysis in general, (3) a range of spectral editing approaches are applied to fully differentiate the various phases solutions (metabolites) through to solids (shell) (4) ^13^C isotopic labelling and multidimensional NMR are combined to provide detailed assignment of metabolites and structural components *in vivo*. While not explicitly studied here the multiphase capabilities of the technique offer future possibilities to study kinetic transfer between phases (*e.g.* nutrient assimilation, contaminant sequestration), molecular binding at interfaces (*e.g.* drug or contaminant binding) and bonding across and between phases (*e.g.* muscle to bone) *in vivo*. Future work will need to focus on decreasing the spinning speed to reduce organism stress during analysis.

## Abstract 2

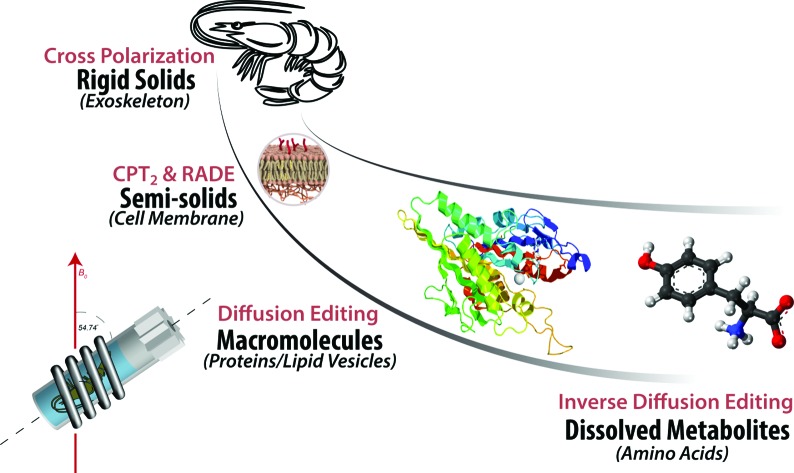
Comprehensive Multiphase NMR provides an overview as to all components (liquids, gels, solids) in a living organism.

## Introduction

Living organisms are defined as systems where metabolism is identifiable. A wide range of environmental factors including physical (temperature, light), chemical (nutrients, drugs, contaminants) and biological (pathogens) can impact metabolism and lead to subsequent disease or change in physiology.^1^ Living organisms encompass a range of materials from soluble metabolites at one extreme, through gel-like components (*e.g.* proteins, muscle, membranes) to true solids (bone, shells) at the other. Changes in shell thickness and structure in the organism can result from environmental and contaminant stress, similarly in human, changes in bone function develop conditions such as osteoporosis^2^ and Paget's disease.^3^ The overall integrating theme is that living systems are multifarious and it is the delicate synergism between physical organization, chemical reactivity, and biological processes that give rise to life. As such, to truly understand biological function and response, analytical tools that can comprehensively study all components within living organisms at the molecular level resolution are desperately required.

Solution-state NMR and MRI have traditionally been used to study living organisms. To date MRI has been arguably the most powerful tool for the study of living systems. Routine MRI studies map water concentrations giving rise to images from which critical diagnoses can be made.^4^ Advanced studies even permit localized spectroscopy which can identify dissolved metabolites. However, there are numerous limitations to MRI based methods which include: (1) only true dissolved molecules can be observed; (2) magnetic susceptibilities lead to broad spectra making identification and quantification challenging; (3) the larger sample volume in MRI systems results in less homogeneous magnetic fields; (4) a lack of lock circuity reduces stability overtime (broad lines); (5) limited spectrometer channels and multinuclear capabilities impede the collection of multinuclear correlation spectroscopy critical for providing spectral dispersion and molecular assignment in complex samples. One simple option is therefore to study living organisms with high resolution solution-state NMR spectrometers. Indeed such studies are highly informative, however, as with MRI, information can only be extracted from the truly dissolved components.^5^ When considering molecular processes (for example, incorporation of dissolved nutrients into solid bone, the crystallization of soluble amyloids to form crystalline fibrils in Alzheimer's, Huntington's and Parkinson's disease) or bioaccumulation of dissolved contaminant or drug within the tissue, the ability to monitor the conversion of one phase into another or the transport across different phase boundaries is essential.

The use of magic angle spinning (MAS) NMR is needed to average chemical shift anisotropy and reduce magnetic susceptibilities in swollen samples permitting both solution-state and swellable (dynamic gels) materials to be investigated. Pioneering studies have been performed showing that organisms can be kept alive under MAS conditions for short periods and high resolution spectral information can be extracted.^6^,^7^ However, all studies to date have utilized High Resolution Magic Angle Spinning probes (HR-MAS) which can only handle low power radio frequency (RF) fields and thus cannot be used to study true solids or rigid gels which require high power decoupling and cross polarization for detection.^8^

In this study comprehensive multiphase NMR (CMP-NMR) spectroscopy, introduced in 2012, is applied for the first time to a living system. CMP-NMR probes contain a lock, pulse field gradients, high power RF circuitry and are fully susceptibility matched.^8^ The result is that all components in all phases can be studied and differentiated *in vivo* and the full range of solution-state, gel-state and solid-state NMR experiments applied without compromise. To demonstrate the proof of principle in this study the organism *Hyalella azteca* (fresh water shrimp) is investigated. One of critically important applications of *in vivo* NMR is the application to understand environmental toxicity and stress.^9^ Traditionally toxicity has been routinely assessed using growth, reproduction rates or mortality as endpoints. However, over time it has been recognized that this approach alone is insufficient and more information regarding toxic mechanisms and the biochemical pathways perturbed is critically needed to explain, how and why specific chemicals are toxic. This need is summarized in a report “Toxicity Testing in the 21st Century” by the National Academy of Sciences (commissioned by the Environmental Protection Agency) which concludes “The new paradigm should facilitate evaluating the susceptibility of different life-stages, understanding the mechanisms by which toxicity occurs, and considering the risks of concurrent, cumulative exposure to multiple and diverse chemicals”.^10^,^11^

An *in vivo* approach with the ability to study all phases would be capable of directly evaluating the mode-of-action, bioaccumulation, biotransformation, molecular reactivity, excretion and binding *in vivo* in response to the organisms surroundings. If specific biological responses can be correlated to certain toxins, then it may eventually be possible to interpret what stressors are truly problematic in a contaminated environment by interpreting the biological fingerprints of the native organisms.

*H. azteca* is considered one of the most sensitive organisms to its environmental conditions and is frequently used and recommended for aquatic and sediment toxicity testing by environmental organizations.^10^,^11^ The organism is used here as a model organism to demonstrate the application of CMP-NMR to study of living organisms in general.

## Results and discussion

### Conditions for survival

**Fig. 1:**
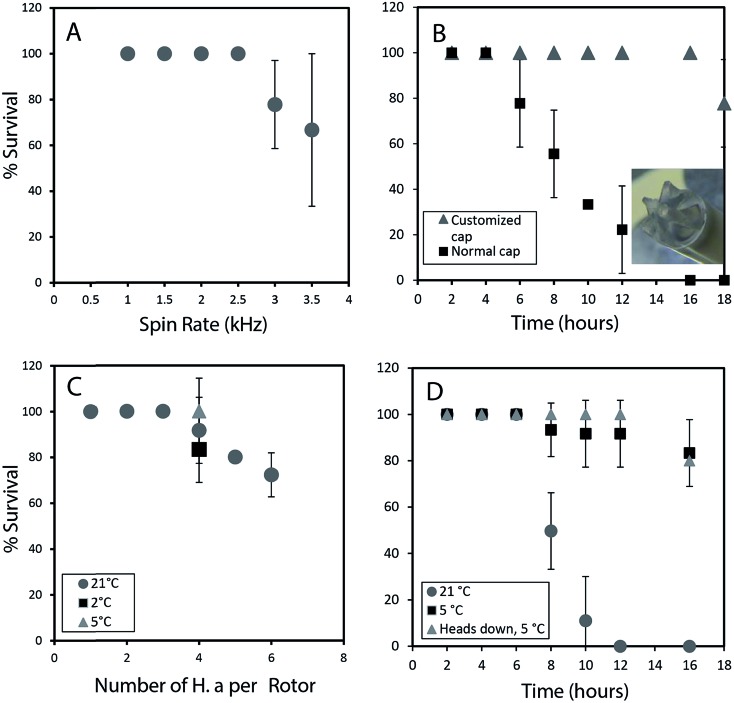
(A) Survival rate in percentage is plotted against several different spin rates (1 h of spinning). (B) The influence of oxygen delivery using a customized cap on improving survival rates is depicted. (C) The effect of loading different number of organisms on the survival is studied. At 5 °C, with lower oxygen consumption survival rates increase to almost 100%. (D) Survival rate up to 16 hours is monitored at different temperatures. When the organism are loaded head first into the rotor there is 100% survival up to 12 h. All experiments were performed in replicates of 3. All studies were performed using the NMR probe external to magnet controlled by a separate variable temperature unit. This permitted use of the NMR spectrometer while these spinning tests were ongoing.

To permit a wide range of NMR experiments required to extract detailed metabolic and structural information, it is imperative that the physical conditions required for *in vivo* magic angle spinning be optimized. The most obvious source of stress is the spinning itself. Fig. 1A depicts the survival rate at various spinning speeds. Numerous repetitions demonstrate that 2.5 kHz is the highest speed that can be used without affecting survival and to ensure the ^1^H spinning sidebands are outside the spectral region using a 500 MHz NMR spectrometer. In future, while considerable additional research is required to reduce spinning sidebands and suppress water, it should be possible to combine slow magic angle spinning approaches such as PHORMAT,^12^ which have been shown effective at spinning speeds as low as 1 Hz and for animals as large as rats to be studied. While successful studies so far have identified a handful of metabolites^12^ a full multiphase approach could provide information on all components within the organisms and low spinning speed should eliminate spinning stress. In this proof of principle study due to complications of sideband and water suppression at low speeds 2.5 kHz spinning was selected as compromise between NMR spectral information and organism stress.

In addition to spinning, oxygen availability was found to be extremely important to the longevity of the organisms. In a conventional NMR rotor the sample is sealed air-tight to prevent the water from leaking. While a wide range of approaches were tested to permit oxygen exchange, the simplest solution was to drill a central hole in the rotor cap and proved to be most effective (inset Fig. 1B). Conventional logic would suggest drilling a hole in the cap would lead to water leakage, however, it was found that while spinning, water pushes against the walls of the seal and a tiny vortex permits air exchange in the center of the rotor. Fig. 1B shows that the use of a modified cap significantly improved the survival rate. Readers should note that Fig. 1B was collected without spinning such that the influence of spinning (Fig. 1A) and oxygen availability (Fig. 1B) can be easily discerned.

The number of organisms per rotor was found to be very important. Tests were carried out with 2–6 week old organisms, and results revealed that *H. azteca* survive the spinning rate at any age. However, packing more than three organisms result in higher mortality (Fig. 1C). The tests were performed using the modified cap and at 2.5 kHz spinning for 1 hour. In a previous *in vivo* study under MAS, anaesthetization was used to improve the recovery rate of the organisms. However, the use of anaesthetic is not ideal as it alters metabolisms and introduces strong signals into the NMR spectra masking key information. As an alternative, here the experiments were performed at a lower temperature. Fig. 1C shows that reducing the temperature to 5 °C is beneficial with a 100% survival rate at 2.5 kHz at this temperature observed (Fig. 1C). To further investigate the effects of temperature organisms were spun at three different temperatures (Fig. 1D) and their survival was monitored overtime. Organisms at 5 °C had a much higher survival rate, in part due to their reduced heart rate and oxygen consumption at lower temperature.^13^ Ultimately, it was found that for the majority of cases spinning at 2.5 kHz for up to 12 hours at 5 °C were good experimental conditions yet often one organism out of 4 would die. Further investigations demonstrated this was caused by evaporation of water through the aeration hole which is accelerated with spinning. The organism closest to the cap would die while organisms lower in the rotor survived. The simplest solution is to use two medium-large organisms or one very large organism and place them with their head down towards the bottom of the rotor.

Throughout this study however, NMR data for single adult shrimp is reported. Fig. S.3† compares the NMR profile for 3 replicates of a single large shrimp (to demonstrate the reproducibility of the approach) with the profile of a single rotor of 3 smaller shrimp for comparison. In general it was found that smaller shrimp would move in an out of the coil while a single larger shrimp always remained within the coil region throughout. In addition, larger numbers of shrimp resulted in a lactate signal appearing in the NMR as a result of anaerobic stress^5^ as-well as alanine, a general stress indicator.^14^ The lactate arises likely due to the higher oxygen consumption from a larger number of organisms, also with more organisms packed into the rotor the organisms on top may be forced up towards the water surface. As such we recommend a single large shrimp be used and placed head down such that the gills remain submerged even if some evaporation occurs.

Fig. 1D shows 100% survival at 2.5 kHz for more than 12 hours at 5 °C. Using larger specimens prevents them from turning around within the rotor allowing their gills to always be submerged. The fact the organisms with their gills submerged survive longer demonstrates the limiting factor (at 2.5 kHz spinning, 5 °C) preventing longer experiments is not the NMR experiments or spinning itself but loss of water due to evaporation from the existing hole in the cap. Future work focusing on air permeable, water impermeable membranes may be able to solve this. ESI (Video 1†) shows the *H. azteca* used to collect the vast majority of data used in the paper. Video 2† shows the *H. azteca* after nearly 14 hours of spinning at 2.5 kHz and 5 °C using the modified cap, 3 weeks following the end of experiments.

### Basic NMR data

#### ^1^H 1D spectroscopy

**Fig. 2:**
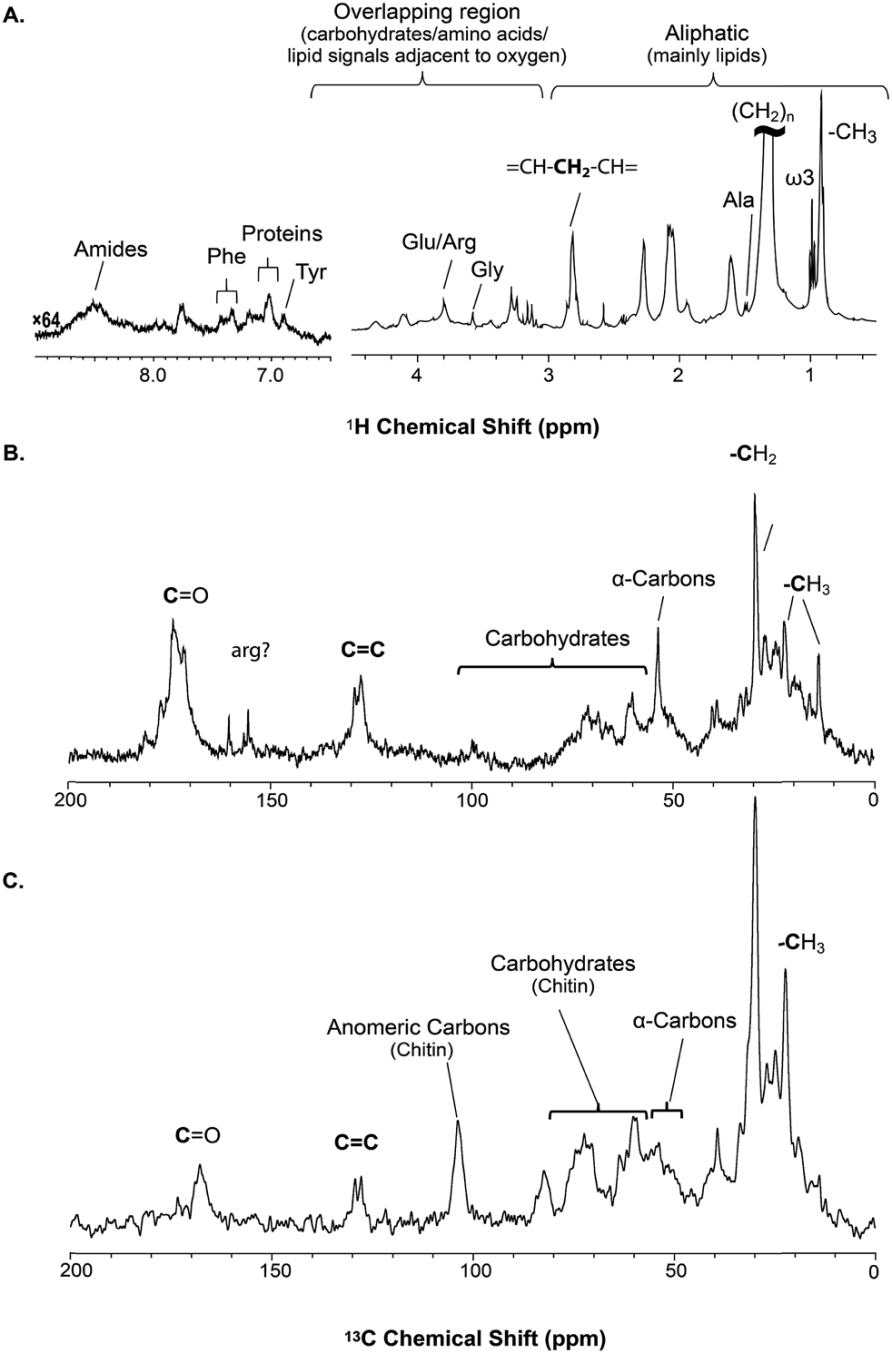
A series of 1D direct NMR experiments are performed, each spectra provides complementary information; (A) ^1^H NMR represents an overview of dynamic components (solutions and gels) in *H. azteca* with overlapping resonances dominated by lipids (B) ^13^C NMR collected with low power ^1^H and provides an overview as to all the carbon in the organism with the true solids suppressed (C) ^1^H-^13^C CP (cross polarization) highlights the solid components in the organisms.

Fig. 2A shows the ^1^H NMR spectrum of a single *H. azteca*. Due to the intense water signal from both the external media and water within the organism, W5 WATERGATE (SPR-W5-WATERGATE) developed for the study of natural samples is employed.^15^ The ^1^H spectrum is dominated by lipid resonances, with protein and metabolite signals also present. As such, the ^1^H NMR data in Fig. 2A represents an overview of the dissolved, gel-like and semi-solid components in the sample, with true solid phases attenuated. ^1^H-^1^H dipolar interactions in true solids can lead to proton line widths of many kHz making them too broad for detection using standard ^1^H NMR approaches.^8^

The solid component, more detailed assignments, and full spectral editing approaches (to separate the solution, gel, semi-solids and solids phase) will be discussed later. The spectrum here was collected using 4096 scans taking 2 h 47 min. Fig. S.1† compares the signal obtained in 256 scans (10.5 min). It is clear that in the aliphatic region the vast majority of information is retained with only 256 scans, but unfortunately in the aromatic region the signal-to-noise is too low for in-depth analysis. As such if relatively concentrated and resolved resonances in the ^1^H spectrum are of interest then collecting real time (every ∼10 min) data should be possible. This however becomes more challenging for metabolites signals buried under the lipid profile which are discussed further in the section “Inverse Diffusion Editing (IDE)”.

#### 1D “high resolution” ^13^C NMR with low power decoupling

Fig. 2B shows the high resolution ^13^C NMR spectrum collected with low power ^1^H decoupling, such that the carbons from the true solids will be discriminated against. The reader can think of the experiment as an overview of all the carbon in the organism albeit with the true solids suppressed (essentially a ^13^C analogue of the ^1^H NMR previously discussed). Using low power decoupling the band width is too narrow to effectively decouple the broad spectral profile of attached protons in the true solid-state.^8^ Note that in this experiment due to the relatively low signal in the carbon experiments, combined with the relatively mobile nature of the carbon detected with low power decoupling, spinning sidebands were not detected and measures to suppress them were not required. As with the proton spectrum, a strong contribution from lipids is characterized by the C=C and (CH_2_)_*n*_ resonances. In addition, contributions from carbohydrates and protein (α-carbons and broad aliphatic distribution 10–40 ppm) are apparent.

#### ^1^H-^13^C cross polarization

^1^H-^13^C CP-MAS of a living organism will strongly bias solid like materials while suppressing mobile components.^8^^13^C CP-MAS is a solid-state NMR technique that transfers magnetization from proton to carbon *via*^1^H-^13^C dipoles. The permanent H–C dipoles which exist in solid structures are ideal for cross-polarization and CP-MAS is very efficient for true solids. However, upon swelling, water introduces local dynamics which modulate the H–C dipolar interactions in turn reducing CP-MAS efficiency. As such the ^1^H-^13^C CP-MAS of a living organism will strongly bias solid like materials including crystalline solids, amorphous solids and rigid-gels.^8^ When applied to the shrimp, strong spinning sidebands were observed consistent with solid material with considerable anisotropy. To suppress the sidebands and permit extraction of the isotropic chemical shift information, total suppression of spinning sidebands (TOSS) was employed.^16^

The ^1^H-^13^C CP-MAS of the shrimp (Fig. 2C) shows strong contributions from lipids, and the carbohydrate chitin (the major component of shrimps shell). These components are further discussed later. When considered holistically, the ^1^H, ^13^C and CP-MAS NMR provide an overview of all the components inside the living *H. azteca* from soluble metabolites to the most rigid solids. However, due to spectral overlap detailed molecular information is challenging to extract. Hence, a series of spectral editing approaches can be extremely useful, in both reducing overlap and providing complimentary information, by highlighting the dissolved, gel, semi-solid, and solid sub-components within the sample.

### Spectral editing

#### Metabolite spectrum (Inverse Diffusion Editing (IDE))

**Fig. 3:**
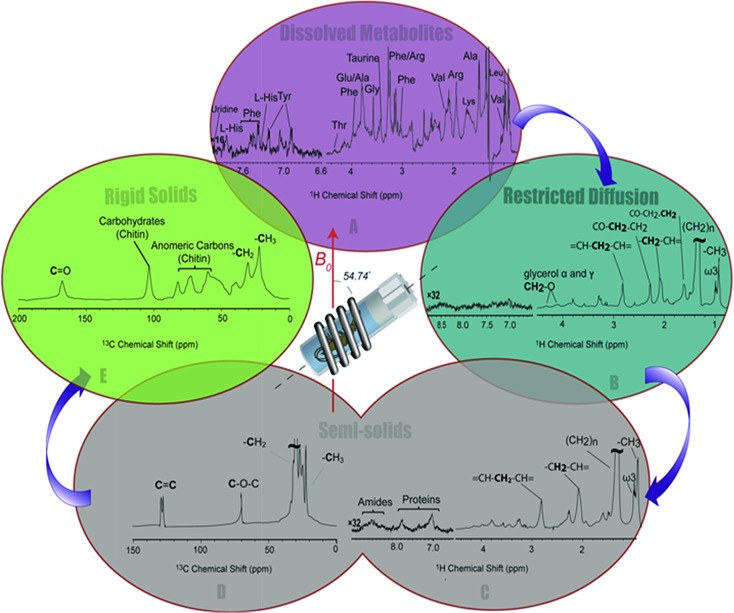
A series of spectral editing approaches applied to differentiate the overlapping resonances are presented. (A) The dissolved metabolites in ^1^H NMR spectra of *H. azteca* are observed in Inversed Diffusion Editing (IDE), (B) gel like metabolites are emphasized in the Diffusion Edited experiments (DE), (C) resonances from the semi-solid components are observed in the ^1^H RADE NMR spectrum, “proteins” refers to aromatic amino acids in macromolecular structures, (D) the semi-solid components are highlighted in ^13^C (CP-*T*_2_) and (E) the most rigid components are selected in the inverse CP-*T*_2_ experiment.

Inverse Diffusion Editing (IDE) selects molecules that are truly in solution and have unrestricted diffusion. IDE is created by subtracting a diffusion edited spectrum (discussed next) from a reference spectrum without diffusion weighting. As such only molecules that move position in space (*i.e.* free to diffuse) are retained and all other molecules are suppressed. In the unedited ^1^H spectrum (Fig. 2A) lipids (many from cell walls, micelles *etc.*) dominate the spectral profile masking key resonances from metabolites, whereas in the IDE spectra (Fig. 3A) they are suppressed. In the IDE spectrum (Fig. 3A) the lipids (which do not show free diffusion) are suppressed and a range of metabolite signals that were previously suppressed can now be fully extracted.^8^ This is critically important as a whole field of research termed metabolomics has evolved, that aims to correlate metabolite response to a range of external variables and stressors.^17^,^18^ Therefore, the ability to extract a high resolution ^1^H profile of just the metabolites *in vivo* is of utmost importance. Unfortunately, however the IDE spectrum does require collecting a diffusion edited spectrum for subtraction which generally has low signal and requires a large number of scans (4096 required for *H. azteca* here). As such this limits the temporal resolution at which IDE can be acquired and makes “real time” flux of these “buried” metabolites signals difficult to obtain. Additional future research into lipid suppression NMR sequences will be required to more effectively extract metabolite profiles in the presence of large overlapping lipid signals.

#### Restricted diffusion (diffusion editing (DE))

Diffusion editing selects components with restricted diffusion. DE involves encoding the spatial position of signals at the start of the experiment and then decodes their position at the end.^19^ The signals from molecules that diffuse are attenuated resulting in a selective spectrum of components that do not undergo diffusion, such as; bound species, large macromolecules, biopolymers, lipid vesicles, *etc.*^8^Fig. 3B shows the DE spectrum for the *H. azteca* which is dominated by lipid signals. These signals also dominate the conventional ^1^H NMR spectrum (Fig. 2A) and likely arise from cell walls or lipids stored mainly in the muscle tissue or hepatopancreas for energy metabolism.^20^ Interestingly, the distinct signal from omega-3 (ω3) fatty acids is present at ∼1 ppm, which was previously identified in NMR spectra of Daphnia^21^ (see later for more discussion). The aromatic region of the diffusion edited spectrum contains few signals with only a very weak broad profile which is consistent with protein resonances.

#### RADE: recovering lost ^1^H NMR semi-solid signals

RADE is an experiment that recovers the loss of relaxation during the diffusion delays and highlights more rigid/semi-solid components such as semi-solid cell membranes, structural proteins *etc.*^8^ Diffusion editing based NMR sequences require relatively long delays during which diffusion is allowed to occur. However, during these delays, molecules with fast relaxation can also return to equilibrium, and if not accounted for these species could go undetected by diffusion based spectral editing approaches. To create a RADE spectrum a control spectrum is collected with all delays and gradient lengths set to zero (essentially the same as a conventional ^1^H NMR spectrum). A second spectrum is then collected with the delays and gradients set as they would be for diffusion editing but the power of the diffusion encoding/decoding gradients set to zero. The difference between the 2 spectra is the signal lost through relaxation which is represented in the RADE spectrum. The RADE spectrum (Fig. 3C) has a strong contribution from lipids similar to the diffusion editing, indicating that along with a range of relatively dynamic lipids (with longer relaxation, appearing in Fig. 3B) a faster relaxing component also exists consistent with more rigid lipids (for example cell walls and less hydrated fat stores). The aromatic region of the RADE shows clear signals from amide and aromatic amino acids from protein. The appearance of protein in RADE and not in DE demonstrates these components undergo fast relaxation consistent with semi-solids and may arise from the muscle (a major protein locale in crustaceans).

#### *T*_2_ filtered CP-MAS (^13^C detected semi-solids)

^1^H RADE presents the most rigid components that can be detected by ^1^H NMR spectroscopy. However, as discussed above, standard “low-power” ^1^H detect experiments will not identify true solids. Cross-polarization (CP) can be used as a filter for the detection of solids *via*^13^C. In previous work we have shown the CP can detect both rigid-gels and true solids, while more dynamic gels and solutions are not observed.^8^ The signals from dynamic solids can be separated from true crystalline solids through the use of a very short (∼30 μs) *T*_2_ filter prior to cross polarization. The result is that only signals from true crystalline solids are supressed while bonds exhibiting dynamics are retained. This spectrum is depicted in Fig. 3D and largely contains lipids, similar to the ^1^H RADE (Fig. 3C). This is expected as previous work has demonstrated that while no signals are missed using the editing schemes outlined here, the components detected by ^1^H RADE and ^13^C *T*_2_ filtered CP-MAS tend to overlap with both techniques observing semi-solids and rigid gel-like materials.^8^

#### Inverse *T*_2_ filtered CP-MAS (true rigid solids)

Inverse *T*_2_ filtered CP-MAS selects only true solids by subtracting the semi-solid component (^13^C *T*_2_ filtered CP-MAS) from the all solid components (conventional ^13^C CP-MAS) leaving the true solids by difference. Fig. 3E shows the rigid solids which are dominated by carbohydrate structures consistent with chitin, the main components of *H. azteca*'s shell.^22^ A proportion of the CH_3_ resonance can also be attributed to chitin. However, the overlap of resonances from ∼55–20 ppm (likely including some of the CH_3_ intensity) also suggests a contribution from rigid proteins that are in the form of chitin–protein fibres or lipid–protein matrices that lie within the cuticles of shrimp and account for the structural strength.^23^

### 2D NMR identification of metabolites

2D NMR provides both additional molecular connectivity information (critical for structural assignment) and affords increased spectral dispersion (reducing spectral overlap). Heteronuclear Single-Quantum Coherence (HSQC) correlates directly bonded ^1^H and ^13^C units and can, in simple terms, be thought of as a high resolution (theoretical peak capacity as a measure of resolution per unit is reported as ∼2 000 000 for HSQC^23^) fingerprint of the H–C framework in a complex mixture. ^1^H-^1^H correlation spectroscopy identifies protons on adjacent carbons. When combined, HSQC identifies the H–C fragments and COSY identifies how these units connect to form a molecule.

**Fig. 4:**
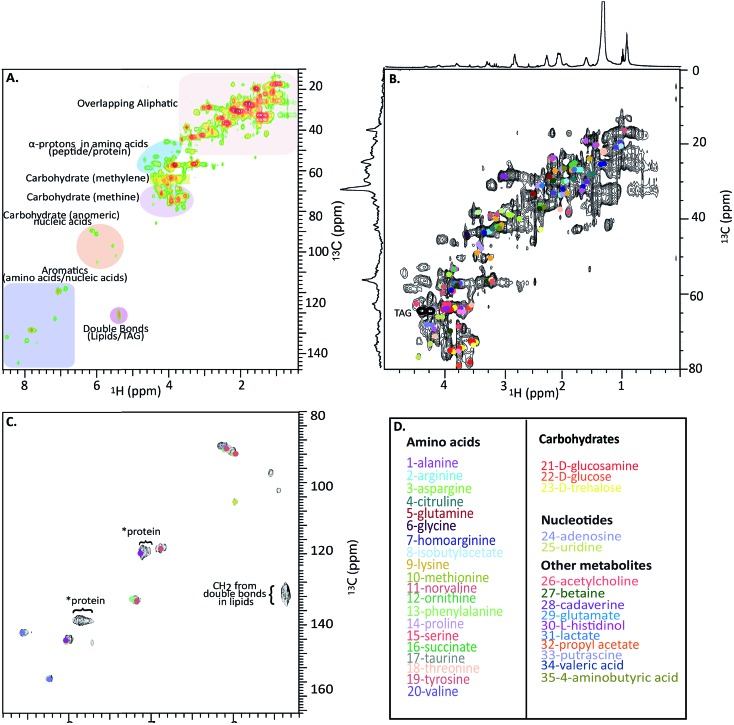
*In vivo* ^1^H-^13^C HSQC of ^13^C enriched *H. azteca*. (A) HSQC with ∼40 metabolites assigned using Bruker's Bio-reference databases. (B) Aliphatic region (C) aromatic region (D) color coded assignments corresponding to the dots in B and C. *These signal dominate the semi-solids spectrum (Fig. 3D) and are consistent with aromatic residues in proteins.

Fig. 4A shows a HSQC spectrum of the *H. azteca* with major regions labelled; aromatics, nucleic acids, carbohydrates, peptides/proteins, lipids and various other aliphatic units. Exact assignments can be made by matching both HSQC and COSY data against bio-reference NMR databases. Approximately 40 metabolites including a range of amino acids, carbohydrates, nucleotides, and other bioactive compounds were identified (Fig. 2B–D and ESI Fig. S.5† for assignment confirmation). The potential role of these compounds as stress biomarkers is addressed bellow. The identification of additional metabolites is challenging, in part due to spectral overlap arising from the complexity of the whole organisms and in part due to limitations of currently available NMR databases. To our knowledge the Bruker Bioreference Databases are one of the most comprehensive metabolites databases commercially available. Still, they only contain 1D and 2D NMR information for ∼650 compounds and no metabolites specific to *H. azteca*. However, a few years ago, there were no commercially NMR databases available and with the rapid evolution of NMR based metabolomics the development of novel databases and open source repositories such as; ; http://mmcd.nmrfam.wisc.edu/ are starting to evolve. With this in mind, careful inspection of the HSQC shows a truly *in vivo* system, this is actually highly encouraging and exemplifies the wealth of information that eventually will be accessible from MAS *in vivo* spectroscopy as assignment resources improve. Further experiments that should facilitate more detailed assignments are considered in ESI.†

### Biological significance

Nearly 40 metabolites were identified in the current study, the most noteworthy of which are discussed briefly here. Close to 20 free amino acids, such as leucine, isoleucine, valine, lysine, and other essential amino acids observed in previous metabolomic studies, appear in the IDE and are confirmed by HSQC.^24^ Other identified metabolites include, lipids, choline, nucleosides, carbohydrates, osmolytes as-well as, structural components such as chitin. For a complete list of the identified metabolites see Fig. 4D where the biological significance of these identified components is discussed next.

Although all amino acids are the basic building blocks of proteins, most amino acids also play major roles in multiple bio-molecular events. The three branched amino acids (leucine, isoleucine and valine) for example are crucial in muscular protein and neurotransmitter synthesis through regulating the mRNA translation.^25^ They can also potentially act as novel biomarkers that may aid in understanding cardiometabolic health.^26^ Corresponding resonances from free tyrosine and phenylalanine are evident in the expanded aromatic region, these two amino acids act as main precursors in many biological processes such as dopamine biosynthesis, which is an intercellular transmitter in multicellular organisms and nervous systems in larger animals.

Aspartic acid, glutamic acid and alanine are found largely as free amino acids mainly in the axoplasm the nervous systems of marine crustacean and account for 20% of the dry weight of the nerves.^27^ Free taurine and glutamic acid may serve as receptors in crustacean antennae and aid in locating the prey in the depth of the ocean or lakes in benthic organisms.^28^ Taurine, glycine, proline, glutamic and alanine may likely be important substances for the regulation of osmotic pressure in the crustaceans muscles.^29^ Other free amino acids are being targeted as potential biomarkers and differentiators of cancers cells^30^ and other disease in general.

The diffusion edited spectra identify an abundance of lipid reserves which were dominated by triacylglycerides (TAG) as major energy storage in most plants and animals.^31^,^32^ The ability to monitor the lipid composition is very important as they are intimately tied to energy metabolism a process commonly perturbed by a wide range of contaminants and other external stressors.^33^ Lipids also serve as a source of essential biomolecules such as hormones. Many invertebrates such as *D. magna* are of particular interest as they cannot synthesize lipids *de novo* and therefore rely on food sources for these molecules.^34^ However, molecular understanding of the process is not well understood and being able to monitor the consumption of food (algae) *in vivo* or examine the transfer of carbon between trophic levels represents a key tool to better understand the ecology and chemistry of food webs. Particularly interesting are the resonances corresponding to ω3 fatty acids in the diffusion edited data, which are an important component of cell membranes as well as precursors to many other substances such as the hormones central to reproduction in many organisms. In addition, the ω3 content of the marine organisms is a “quality indicator” in the food industry, since they are the primary source of ω3 for human consumption and cannot be biosynthesized in mammals.^35^

The diamines putrescine (1,4-diaminobutane), cadaverine (1,5-diaminopentane)^36^ produced by the deamination of lysine or ornithine^37^ are found in high abundance in all major groups of marine organisms such as invertebrates. They are naturally occurring polyamines, which regulate the concentrations of the cations in macromolecular structure of DNA and RNA or their transport through cellular membranes.

The nucleosides adenosine and uridine, represent important biomarkers related to DNA/RNA damage, which have been implicated to be an indication of carcinogenesis. DNA/RNA monitoring can act as a measure of hereditary risk of cancer or many other diseases.^38^ Choline is another identified metabolite that appears in the head groups of some phospholipids. It is also a precursor needed to form acetylcholine which is a neurotransmitter that controls memory and muscle movements. Therefore, choline plays major part in many nervous system related disorders.^39^ Also, fluctuation in choline level is a well-established biomarker for different cancer types.^30^

Chitin is the main structural polysaccharide that forms the protective exoskeleton in all arthropods, which makes up to 60% of their mass and serves as an important structural component in their body. It is periodically shed and causes their growth in several stages of molting.^40^,^41^ Several steps take place to convert trehalose to chitin in which mainly glucose, glutamine and uridine triphosphate (UTP) serve as precursors of its synthesis. The presence of most of the precursors and products through these stages are identified in the 2D spectra. The ability to study soluble precursors, through conversion into a gel and finally a solid shell are only possible using a CMP-NMR probe where all phases can be studied and differentiated *in situ*. The approach opens up exciting possibilities to follow process such as bone formation. Indeed not just the formation but the degradation of the solid materials could be an important complimentary source of information^42^ itself key to stress induced thinning in bird egg shells and shells of marine animals, as well as the degradation of bone with age.

### Towards *in vivo* organism monitoring using CMP-NMR

The major drawback of *in vivo* CMP-NMR is the potential stress from the spinning itself. Fig. S.2† compares the ^1^H NMR over a 20 hour period of continuous spinning at 2.5 kHz. In this case a large single shrimp was used and the organism was still alive after 20 hours of spinning. The main ^1^H NMR profile remains consistent. The main difference is the appearance of alanine, which has been reported as a general indicator of stress.^14^ The lack of lactate (an indicator of anaerobic stress) observed when numerous organisms are placed in a rotor (see Fig. S.3D†), suggests that spinning rate (rather than lack of oxygen) likely plays a role in the alanine production. While it can be argued that moving forward, spinning stress could be reduced (*e.g.* lower spinning rates) eliminating it completely may never be possible. As such it is important to discuss potential ways in which this stress could be accounted and the best way to incorporate CMP-NMR into an *in vivo* monitoring program. This can be achieved through using CMP-NMR as a stand-alone tool or as part of a larger *in vivo* NMR framework, the approaches will be discussed briefly and separately.

### Using CMP-NMR as a part of a larger *in vivo* NMR framework

Recently our group has demonstrated that flow based *in vivo* NMR is an excellent low-stress approach to understand metabolic change.^5^ Organisms are placed in a NMR flow tube (which is kept static) and oxygenated water (with or without food) is continually recirculated. Contaminants, stressors or nutrients can be easily introduced into the flow making it ideal for monitoring metabolic response to stress. The drawback however is the low resolution from the ^1^H NMR spectra. While this can be overcome *via* spectral dispersion afforded by ^1^H-^13^C HSQC, information is still only obtained for the truly dissolved metabolites. Conversely, in CMP-NMR the NMR profile is sharpened through MAS and information on the gels and solids can also be obtained. CMP-NMR would be best employed at time point when flow-NMR identifies responses and the additional capabilities of CMP-NMR used to provide additional resolution to aid with metabolite assignment as-well as provide information on gels and solids only detectable by CMP-NMR. In this way CMP-NMR plays more of a supporting role in terms of metabolic change, and a primary tool for observing structural change. This approach leverages the low stress attributes of flow NMR to monitor metabolic change while benefitting from CMP-NMR for assignment. In such a combined approach as conclusions regarding stress fluxes are drawn from the low stress technique, additional stress from the spinning in CMP-NMR becomes less critical.

### CMP-NMR as a stand-alone tool

While the combined approach would be recommended for large studies aimed at truly targeting the metabolic stress, there are situations where the approach may not be feasible or available. In such a scenario spinning stress would have to be differentiated from impacts of exposure (contaminant, drug *etc.*) *via* controls. The controls should be identical to the dosed organisms with the exception of the addition of the stressor. In its simplest form differences after chemometrics analysis between the control and dosed organisms, spun for the same amount of time, should arise solely from the stressor as stress arising from spinning should cancel. Such studies should be carefully controlled in terms of spinning time and if multiple NMR experiments are performed they should be run in the same order and started at the same time after spin initiation on both the dosed and control subjects such that spinning effects cancel. As the stress from spinning likely manifests over time (evidenced by the increase in alanine in Fig. S.2†) more advanced studies could use time based chemometric trajectory analysis to further differentiate metabolites co-involved in the separate processes.^43^ Of particular interest may be to introduce the chemical stressor midway the analysis such that trajectory from spinning remains linear throughout while the influence of the stressor impacts only the later datasets.^44^ Currently our group is working toward protocols for using CMP-NMR effectively to understand stress processes. Given the unique and comprehensive *in vivo* information that CMP-NMR can provide over more traditional NMR and other non-NMR based methods stress from spinning is an unavoidable side-effect that must be accounted for.

## Experimental

Results (Fig. 1) suggest that *H. azteca* spun at a reasonably slow rate 2.5 kHz are limited by the oxygen content in approximately 80 μL of water in the rotor, but are not limited by the spinning itself. To minimize this oxidative stress a hole is drilled into the cap. Rotor caps with o-ring seals were purchased from Wilmad Glass and drilled with a 0.022′′ inch drill bit to produce a hole allowing for air exchange thereby extending the survival of *H. azteca* under MAS conditions. Viability studies were performed in triplicate and the percentage survival rate along with the standard deviation were reported (see Fig. 1).

The original *Hyallela azteca* culture was provided by Environment Canada from their main colony. The organisms were then cultured within the University of Toronto laboratory and living conditions for the specimen were controlled according to methodologies by Environment Canada^10^ and kept similar to their natural habitat in fresh water. They were kept in 20 L tanks with a 2 cm layer of sand in dechlorinated, aged tap water, continuously aerated as their medium. 20% of the overlaying water was changed 3 times a week before feeding time. The tanks were exposed to 16 : 8 h light to dark photoperiod using a fluorescence commercial lamp and the temperature was kept at constant 24 °C. Carbonate hardness of 124 mg CaCO_3_ L1 (consistent with local freshwater conditions). ^13^C isotopically labelled *Chlamydomonas reinhardtii* were cultivated in a small scale closed loop system. A custom photobioreactor built by Silantes GmbH was used for production of algae biomass. Each fermentation has been conducted autotrophically and exclusively with ^13^CO_2_ (98% enriched with stable isotope ^13^C). The cultivation parameters including media, temperature, light intensity, and pH were adjusted to gain a maximum growth rate. The harvested algal biomass was fed at a rate of 2 mg per *H. azteca*, 3 times a week over their life span and represents their sole carbon source. For more details regarding the culturing of the isotopically enriched algae please refer to ESI.†

A 4 mm zirconium rotor was filled with water. For offline viability studies (Fig. 1) 1-6 *H. azteca* were used. The *H. azteca* were selected based on size with <2 mm representing smaller shrimp, 2–5 mm medium shrimp and >5 mm large shrimp. Size was gauged using a microscope and males were selected for analysis. Different sexes can be recognized by the absence of an enlarged gnathopod or presence of egg case in females. For our experimental purpose adult males were selected due to large body size as well as to avoid the inconsistencies due to additional lipid storage within the eggs in the females. For all the NMR data reported here (with the exception of Fig. S.3D†) a single large shrimp (length ∼ 7 mm) was used and placed in the rotor with its gills towards the bottom. *H. azteca* were loaded from the droplet at the tip of a plastic pipette and let swim into the rotor (refer to Video S.1 in the ESI†). A trace of D_2_O (∼3 μL) was added to act as a spectrometer lock. The rotor was sealed using a top Kel-F cap with an o-ring seal customized with a hole as described above. All NMR spectra were acquired using a Bruker Avance 500 MHz Bruker Avance III Spectrometer at a spinning rate of 2.5 kHz fitted with a prototype CMP MAS 4 mm ^1^H-^13^C-^2^H probe with an actively shielded magic angle gradient (Bruker BioSpin). All experiment were performed at 5 °C and locked on D_2_O, sample temperature was not seen to increase more than 1 °C during any experiments. The lock was maintained for all experiments including solids experiments. After each run the organism was monitored for 1 week before being returned to the main colony. Only NMR data from organisms that survived and fully recovered were used in this paper.

Additional information including total experiment time, *T*_1_ values and delays used are provided in Table S.1.† ^1^H NMR SPR-W5 WATERGATE pulse sequence was used for water suppression^15^,^45^ with garp-4 decoupling to remove ^13^C splitting. The shaped pre-saturation prior to W5 was achieved using a train of selective pulses: 1000, 2 ms, calibrated rectangular pulses were used, each separated by a 4 μs delay. 256–4096 scans (see Table S.1†) were collected with 8192 time domain points, 20 ppm sweep width, a 125 μs binomial delay and a recycle delay 5 × *T*_1_. Spectra were processed using an exponential function corresponding to a line broadening of 2 Hz in the transformed spectrum and a filling factor of 2. Carbon spectra were obtained using spectral width of 400 ppm, 16 384 time domain points, 5000 scans inverse gated decoupling (Waltz-16) and a recycle delay 5 × *T*_1_. Spectra were processed using an exponential function corresponding to a line broadening of 5 Hz in the transformed spectrum and a filling factor of 2.

^1^H diffusion based editing was performed with a bipolar pulse pair longitudinal encode-decode (BPPLED) sequence.^19^ Scans were collected using an encoding/decoding gradients of 1.8 ms at ∼50 gauss per cm (65 gauss per cm max for current probe) and a diffusion time of 180 ms. Inverse diffusion editing (IDE), relaxation Recovery Arising from Diffusion Editing (RADE) and inverse *T*_2_ filtered ^13^C CP/MAS were done by appropriate spectral subtraction as previously described.^8^ Briefly, the spectra are scaled until the spectra being subtracted was nulled with the resulting difference spectra containing positive peaks.

CP/MAS was performed using linear ramp defined by 1000 points from 80–100% during a contact time of 2 ms, high power composite pulse decoupling (Spinal 64) and Total Suppression of Sidebands (TOSS).^16^ Decoupling was applied using an RF field of 50 kHz, improvements were not seen beyond this as such the RF field strength was applied at 50 kHz to prevent additional sample heating. 30 720 scans were collected using 1024 points and a recycle delay of ∼5 × *T*_1_ (^1^H macromolecules, see Table S.1†). Spectra were processed using an exponential function corresponding to a line broadening of 25 Hz in the transformed spectrum and a filling factor of 2. For the selection of semi-solids (CP-*T*_2_) a train of 2 × 7.5 μs CPMG echoes were applied to the ^1^H spins prior to contact.^8^ The inverse CP-*T*_2_ (solids) was created by difference. Singular-value decomposition^46^ was applied to CP-*T*_2_ and ΔCP to reduce spectral noise (an example of which is shown in ESI Fig. S.4†).

^1^H-^13^C HSQC (Heteronuclear Single-Quantum Coherence) spectra were collected in phase sensitive mode using Echo/Antiecho-TPPI gradient selection, with 600 transient for each of the 128 increments in the F1 dimension. 2048 time domain points were collected in F2 and a 1J ^1^H-^13^C of 145 Hz. F2 was processed using an exponential function corresponding to line broadening of 15 Hz and F1 using sine-squared functions with a π/2 phase shift. 2D COSY (correlation spectroscopy) spectrum was acquired to confirm HSQC assignments of metabolites. The COSY experiments were collected using an in-phase approach,^47^ using gradients for coherence selection and low power ^13^C garp decoupling throughout. 128 scans and 2048 data points were collected for each of the 196 increments in the F1. Both dimensions were processed using sine-squared functions shifted by 90°, zero filling factor of 2. Compound identification and quantification were performed using AMIX (Analysis of MIXtures software package, version 3.9.15, Bruker BioSpin, in combination with the Bruker Bioreference NMR databases, versions 2-0-0 to 2-0-4) as previously reported.^48^ Only assignments with an *R*^2^ correlation >0.99 between the observed and databases shifts were retained. Where possible correlations were also confirmed with COSY.

## Conclusions

In this study the main objective was to demonstrate the feasibility and applicability of CMP-NMR to a whole living organism, while retaining the biological function. The study demonstrates all structural components can be identified, from metabolites to true solids. The ability to monitor potential real-time molecular fluxes and relate this directly to biological response, activity or behavior provides an essential link to better understand biological processes and ultimately the biochemistry and chemistry that define these events. The potential of the current work goes way beyond the freshwater shrimp studied here and could theoretically be applied to a wide range of small organisms, complete tissue sections (including bone), and to follow almost any biological process, including, growth, reproduction, disease, stress, and others. To our knowledge, this approach arguably provides the greatest amount of molecular information *in vivo* compared to any modern scientific tool, and the versatility to extract information from all components, solutions, gels and solids, is quite profound. Of course the approach does not come without limitations, some of which are discussed and addressed in ESI† section.

In summary, this study demonstrates it is possible to study a whole living organism by CMP-NMR. The unique capabilities afford the possibilities to study and differentiate all phases (solutions, gels, semi-solids, true solids) *in vivo*. Theoretically, this provides the potential to study all organic components within a living organism, providing an unprecedented window into biological processes and stress responses. Considering the huge potential along with the unique and unprecedented molecular information the approach affords, it is clear *in vivo* CMP-NMR will have an important role to play in many areas of research. The technique acts as the ideal “molecular interpreter” providing the desperately needed connection between the physical (for example environmental stress and disease) to the chemistry that ultimately defines these processes.

## Supplementary Material

Supplementary information
