# Supplementary material for: Comprehensive multiphase NMR applied to a living organism
Source: Chem Sci. 2016 Apr 18;7(8):4856–66. doi: 10.1039/c6sc00329j (PMC6016732; doi:10.1039/c6sc00329j)
Supplement: Supplementary file 1 [file SC-007-C6SC00329J-s001.pdf]

Electronic Supplementary Material (ESI) for Chemical Science.
This journal is © The Royal Society of Chemistry 2016

Comprehensive Multiphase NMR Applied to a Living Organism

Yalda Liaghati Mobarhan[1], Blythe Fortier-McGill[1], Ronald Soong[1], Werner E. Maas[2], Michael Fey[2], Martine Monette[3], Henry J. Stronks[3,] Sebastian Schmidt[4], Hermann Heumann[4] Warren Norwood[5] and André J. Simpson[1*]

(1) *Department of Physical and Environmental Science, University of Toronto, 1265 Military Trail, Toronto, ON, Canada, M1C 1A4*
(2) *Bruker BioSpin Corp., 15 Fortune Drive, Billerica, Massachusetts, USA, 01821-3991*
(3) *Bruker BioSpin Canada, 555 Steeles Avenue East, Milton, ON, Canada, L9T 1Y6*
(4) *Silantes GmbH, München, Germany*
(5) *Environment Canada*, 867 Lakeshore Rd., *Burlington,* ON, Canada L7R 4A6

***Corresponding Author Information:**
André J. Simpson
Department of Chemistry
University of Toronto Scarborough
1265 Military Trail
Toronto, Ontario.
Canada, M1C 1A4
Telephone: 1+(416)-287-7547
Email: andre.simpson@utoronto.ca

Supporting Section Index

Part A. Supporting Discussion and Methods (page 2)

Part B. Supporting Figures and Table (page 3)
Figure S.1
Figure S.2
Figure S.3
Figure S.4
Figure S.5
Table S.1

Part C. Supporting Videos (page 8)
Video S.1
Video S.2

References (page 8)

Supporting Section

Part A. Supporting Discussion and Methods

It has been demonstrated that the organisms could be kept alive and fully recover from up to 14 hours of spinning at 2.5 kHz at 5°C. However, while this simplifies the NMR aspects of the study, the process itself will likely induce some stress. Techniques developed for slow magic angle spinning such as PHORMAT[1] can theoretically be applied and should eventually permit the extraction of chemical shift information, at spinning speeds as low as 1 Hz, and permit animals potentially as large as rats to be studied.

Interpretation of the spectra at present is challenging, as discussed previously this is in part due to the fact that NMR databases are still somewhat in their infancy, albeit they are evolving at a rapid rate in parallel with the growth of NMR based metabolomics in general. The main remaining hurdle then becomes the spectral overlap which hampers both interpretation and subsequent quantification. The easiest quantitative information could be accessed from the basic 1D NMR in combination with Electronic REference To access In vivo Concentrations (ERETIC), a method developed to provide quantification from MRI, without the need for internal standards[2]. Unfortunately the overlap in basic 1D NMR makes this challenging for most species. Theoretically quantification can be done via the spectral editing approaches (Figure 3), but additional ERETIC based protocols will be required that account for signal fraction during the weighted subtractions required for editing. ERETIC is possible in multidimensional NMR,[3] and when combined with special data acquisition techniques can be completely quantitative[4]. The sub-fraction of signals in the multidimensional dataset can be related to the total signal from the whole organism, which can theoretically be achieved through spin counting[5]. While considerable research in this area is required, given that NMR (if acquired appropriately) is highly quantitative and that the fundamental tools to quantify *in vivo* without standards already exist, providing absolute quantitation should be feasible in the near future. As such the complexity of the whole organisms and the resulting spectral overlap remains the key challenge. One of the easiest way to further reduce overlap (along with providing more connectivity information for assignment) would be to integrate other heteronuclear information into the NMR experiments to further provide spectral dispersion. Consider for example the peak capacity of a ^{1}H-^{13}C HSQC dataset (employed here) is generally considered to be ~2,000,0000, while the peak capacity of 3D NMR is ~100,000,000[6]. The most obvious choice would be the incorporation of both a ^{13}C and ^{15}N isotopic label into the food source (i.e. ^{13}C/^{15}N doubled labelled algae), giving rise to double labelled organisms. In this case, the great wealth of experiments to study biomolecules can be applied and it would be relatively easy to use ^{15}N editing to extract sub-spectra that contain only amino acids, or even spectra for specific amino acids or target metabolites. ^{31}P would be complimentary and could provide direct evidence relating to nucleotide bases and key energy molecules such as ATP and ADP. ^{14}Si represents a very important nucleus with diatoms representing key contributors to the global fixation of carbon. A recent review concluded that the lack of molecular knowledge regarding silicon sequestration into diatoms (the major limiting nutrient in diatom growth) is limiting progress in this field of research[7]. The ability of the CMP probe to study all phases would allow researchers to follow how soluble silica is absorbed, transported by organic molecules and precipitated as solid cell walls *in vivo*. Beyond structure elucidation, *in vivo* CMP-NMR is ideal to study molecular interactions. It has the ability to follow *in vivo* binding, transformation, sequestration, bioaccumulation and excretion of the contaminants and drugs. In this light ^{19}F common in many pharmaceuticals and environmental contaminants[8] provides a unique handle to selective view xenobiotics, and to elucidate their dynamics and kinetics[9, 10]. If ^{19}F is not naturally present ^{2}H provides the potential to introduce an NMR active nucleus into pretty much any organic structure with little alteration to the molecular chemistry[11].

Culturing of the isotopically labelled algae

^{13}C,^{15}N-labeling of the algae *Chlorella reinhardtii* (wt, SAG culture collection Goettingen, Germany) was achieved by growing the cells in $^{13}CO_2$ and $^{15}NH_4Cl$ (enrichment in both cases 99%; purchased from Sigma Aldrich Isotec, St Louis, Missouri) for 150 hours in a photobioreactor(PBR) constructed by Silantes GmbH (Munich Germany). The PBR is built as a closed system avoiding loss of the stable isotope labeled ^{13}CO2. It is an airlift driven external loop tubular fermenter having 20 L operating volume, permitting circulation of the media volume with 1 vvm. The algae were cultivated in regular TP media (Tris-Phosphate w/o Acetate + $^{15}NH_4Cl$), as previously described[12], using the following parameters: pH 7.0 - 7.4 (pH-probe InPro®3253I/Sg, Mettler Toledo), temperature 30°C (InPro®3253I),

light intensity 1300 µmol/(m^2·s) (quantum detector LI-250A, Li-Cor Biosciences GmbH). A computer-controlled gas management system (Labview 10.1, National Instruments) was incorporated to keep the $^{13}CO_2$ content (pCO_2-probe InPro®5000,Mettler Toledo) at a concentration of 2%. The nitrogen carrier gas was allowed to vary between 65% and 85% corresponding to the oxygen content which increased during autotrophic growth of algae between 15% and 30%. If the oxygen content reached a value of 30% (pO_2-probe Visiferm Do Arc., Hamilton) an N_2 purging step was introduced in order to reduce the O_2 content to the initial value of 15%. To avoid loss of ^{12} CO_2 during N^2 purging, the $^{13}CO_2$ addition was stopped at the end of a growth cycle until a value of 0.5% was reached by metabolic depletion. If the oxygen reached the initial value of 15% due to N_2-purging, the computer controlled valves (Valve 221606, Buerkert) were closed and another growth cycle was resumed. The ^{13}C-content of the algae biomass was determined by analyzing the enrichment in "C_{18}" fatty acids of isolated algae biomass by GCMS (Thermo Quest Polaris Q MS / Trace GC2000, Thermo Fisher). The ^{15}N-content was determined by Shanghai Research Institute of Chemical Industry stable isotope laboratory. Isotopic enrichments of 98-99% ^{13}C and ^{15}N were achieved in the algae biomass.

Part B. Supporting Figures and Table

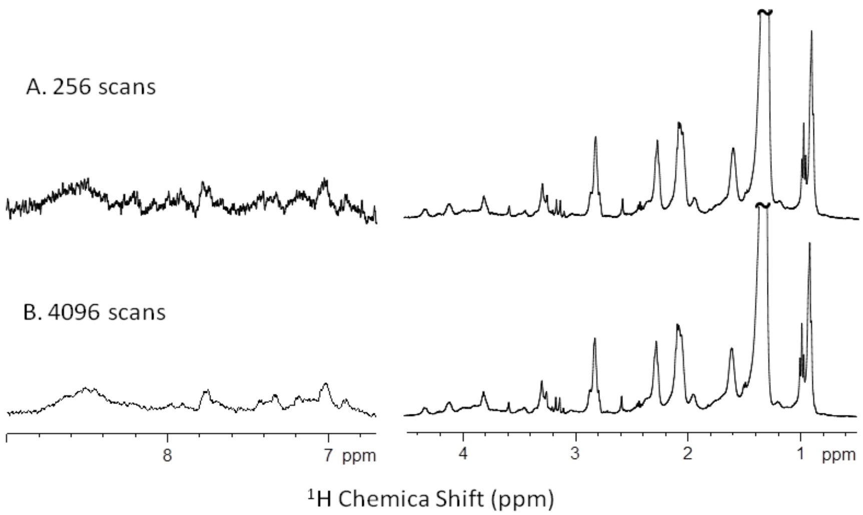

Figure S.1 ^1H NMR data collected for a single shrimp using A. 256 and B. 4096 scans. While little information is lost in the aliphatic region, the lower signal-to-noise in the aromatic region reduces the information content in this region.

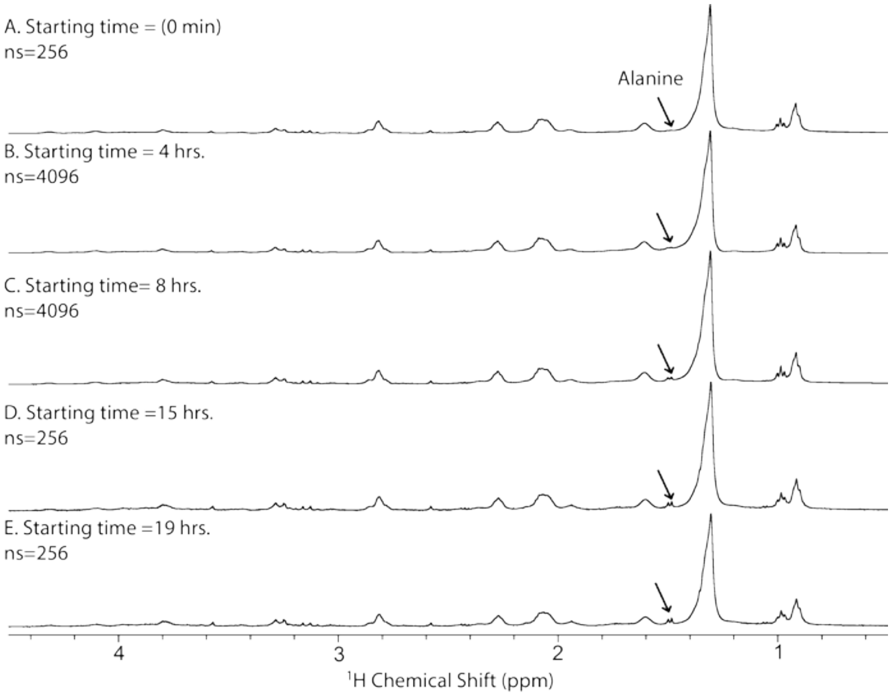

Figure S.2 ^{1}H Spectra acquired on a single shrimp over a 20 hour period. Note spectra A, D, E were collected using 256 scans, and spectra B and C using 4096 scans. The alanine signal increases over the 20 hour period and may arise from spinning stress.

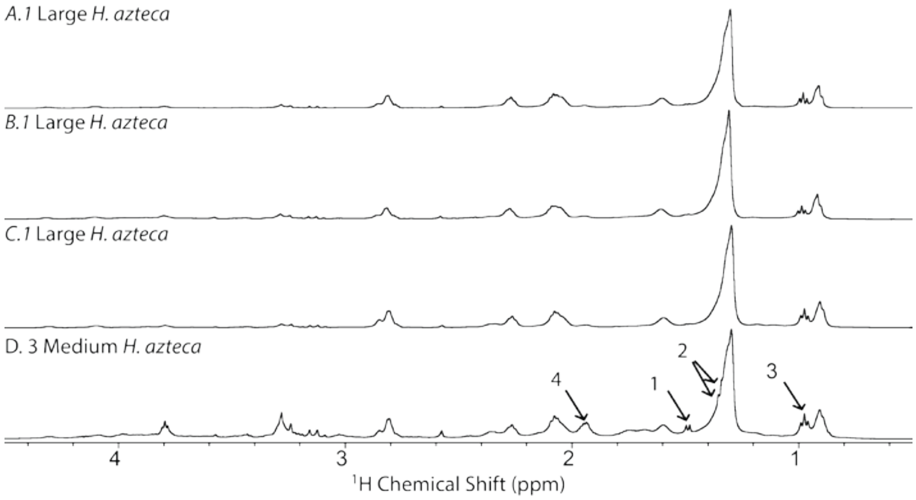

Figure S.3 A-C compares the spectra from 3 different adult *H. azteca* (~7 mm length each). The spectral profiles are similar indicating the approach has a good degree of reproducibility. In contrast panel D shows the spectrum for 3 medium sized *H. azteca* (~3 mm length each) placed in the same rotor. A contribution from the doublet of lactate (2) on the left shoulder of the $(CH_2)_n$ resonance is an indication of anaerobic stress[13]. While alanine (1) has been reported as an indicator of stress in general in aquatic organisms[14]. Interestingly these younger *H. azteca* have strong contribution in regions (3) and (4) which have been assigned to omega-3's and an overlapping region where DHA (docosahexaenoic acid) resonates[15]. This would be consistent with these compounds being essential to

invertebrate early growth[16] and indicates that CMP-NMR could be a useful tool in general to study chemical physiology and dynamics.

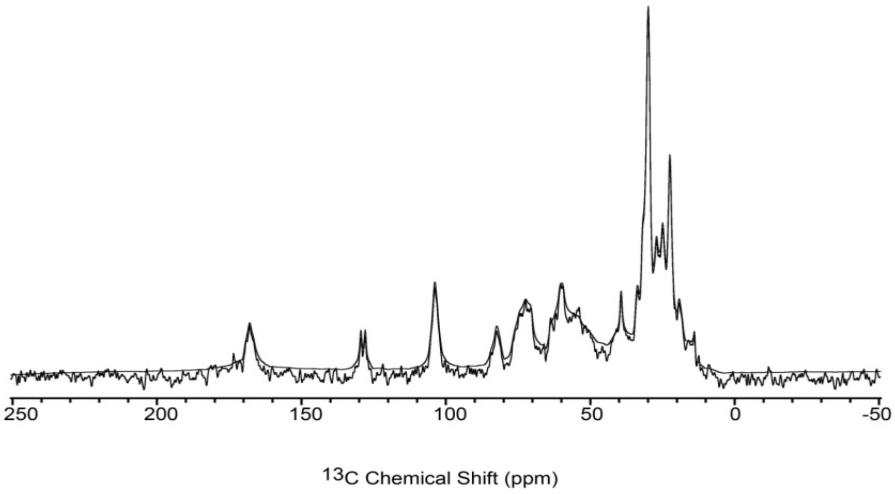

Figure S.4 Overlaid spectra of a ^{1}H–^{13}C CP before (solid line) and after (dotted line) denoising using Singular-value decomposition.

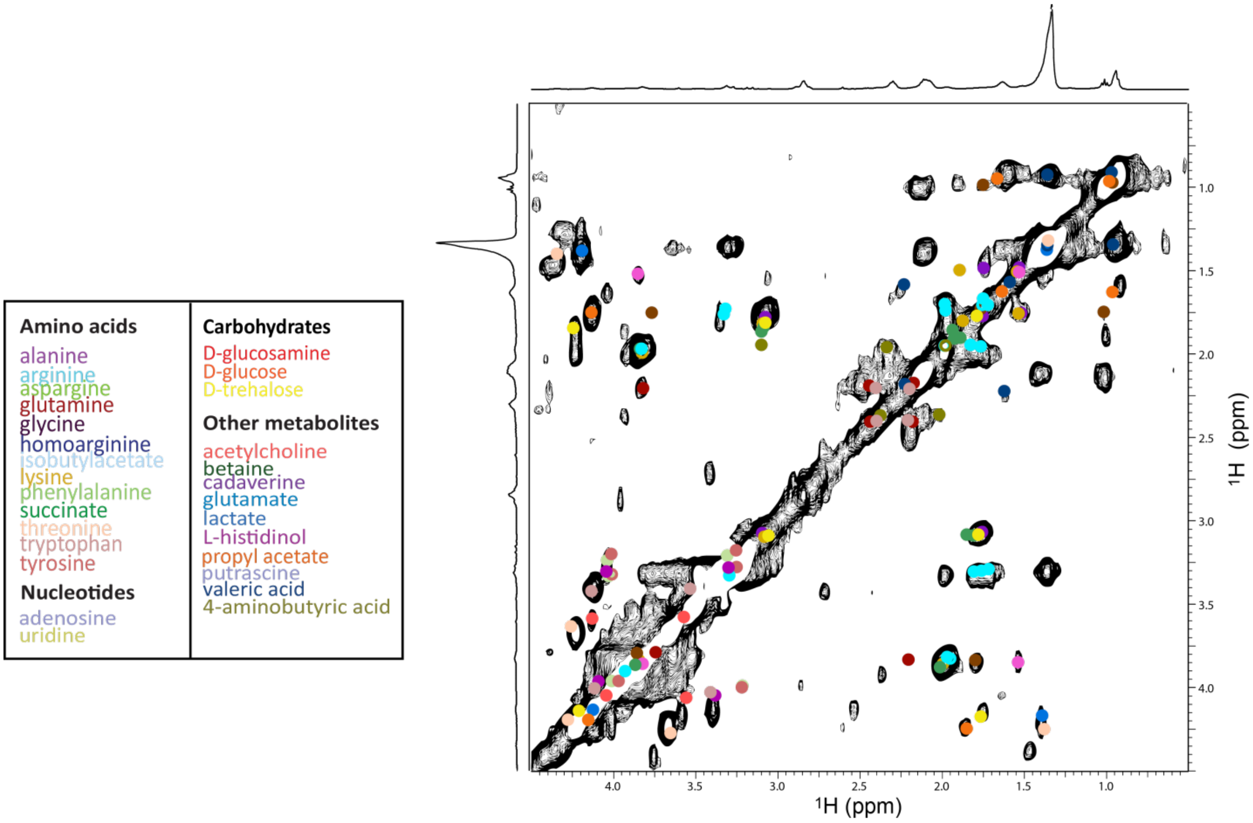

Figure S.5 2D In-Phase-COSY experiment used to further confirm the assignments reported in the main section of the paper.

	Experiment	Sample	Rep. Rate	NS	Experiment Time	Comment
**Run 1**	**^{1}H**<br>**^{1}H T_2**<br>**Diffusion Ref.** **(no delays /no gradient)**<br>**Delays/no gradient (needed for RADE)**<br>**Delays and gradient on (diffusion editing)**	1 Large shrimp	2.4 s	256* or<br>4096<br>4096<br>4096<br>4096	10.5 min or 2 hrs. 47 mins<br>2 hrs. 47 min<br>2 hrs. 47 min<br>2 hrs. 47 min<br>2 hrs. 47 min	The longest ^{1}H T_1 for metabolites were estimated at 400 ms (inverse recovery experiment) leading to a repetition rate of 2.4 s (2 s delay + 0.4 s acquisition time). The relatively short relaxation times arise due to the fact the sample is a whole organism and it is uniformly ^{13}C labelled (^{1}H relax via ^{13}C interactions).<br><br>Due to the lower signal in diffusion editing 4096 scans were required in this experiment. For consistency 4096 scans were also collected for the other ^{1}H data shown in the main paper. However, fewer scans still provide useful data.<br>*See Figure S.1 which compares the signal from 256 scans (10.5 mins) with that from 4096 scans (2hrs 47 mins).
**Run 2**	**^{13}C (zgig)**	1 Large Shrimp	5.16 s	5000	7 hrs. 13 min	The longest ^{13}C T_1 for metabolites were estimated at 1 s leading to a repetition rate of 5.16 s (5 s delay + 0.16 s acquisition time). The relatively short relaxation times arise due to the fact the sample is a whole organism and it is uniformly ^{13}C labelled (^{13}C relax via ^{13}C interactions).
	**CP**<br>**CPT_2**		0.5 s	30720<br>30720	4 hrs. 27 min<br>4 hrs. 27 min	Cross Polarization is only efficient for semi-solids and solids, dissolved components do not undergo CP[17]. Inversion recovery showed a sharp metabolite profile superimposed over a broad macromolecular background. The background rapidly relaxed exhibiting a T_1 estimated at 100ms. As such the recycle delay was set at ~ 5 x T_1 of the macromolecular background (or ~1.2 times the metabolite T1's). Readers should note however, that CP-MAS is not considered fully quantitative and is only used for editing purposes in this manuscript.
**Run 3**	**COSY**	1 Large Shrimp	0.5 s	NS=128<br>Increments =196	6 hrs. 42 min	As COSY is not fully quantitative the recycle delay was set at ~1.2 T_1 for max signal per unit time[18].
**Run 4**	**HSQC**	1 Large Shrimp	0.5 s	NS=600<br>Increments =128	14 hrs. 41 min	As HSQC is not fully quantitative the recycle delay was set at ~1.2 T_1 for max signal per unit time[18].

Table S.1 Summary of experimental runtimes and associated parameters.

Part C. Supporting Videos

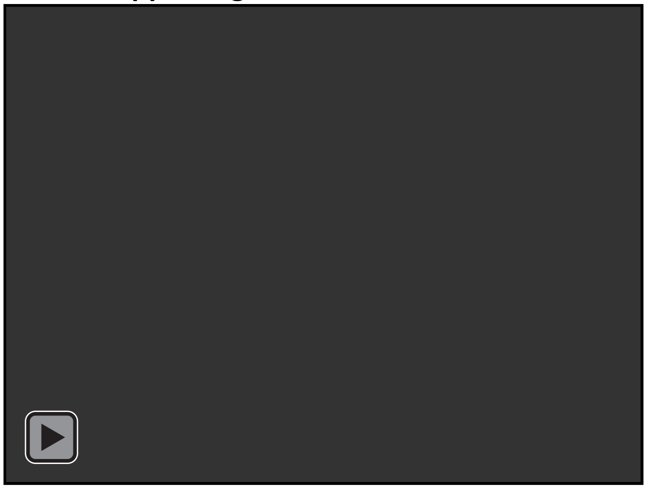

Video S.1 Loading a large adult *H. azteca* heads in first, into a sapphire rotor.

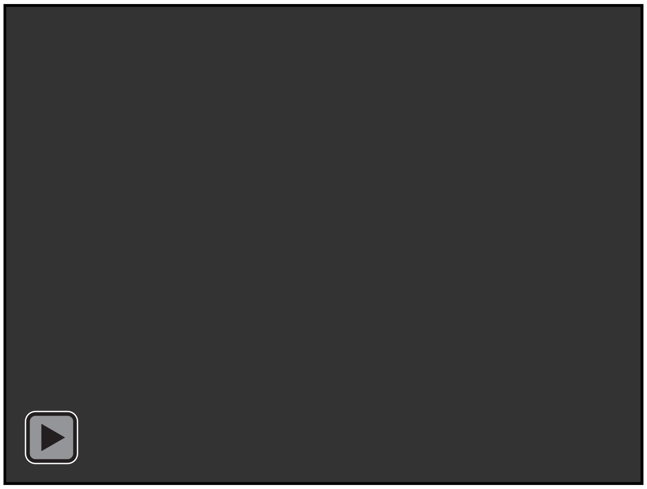

Video S.2 Showing the *H. azteca used to collect the majority of data for the paper 3 weeks* after nearly to 14 hours of MAS (2.5 KHz, modified cap, 5 ^{o}C) NMR experiments

References

1. J. Z. Hu, W. Wang, F. Liu, M. S. Solum, D. W. Alderman, R. J. Pugmire and D. M. Grant, *Journal of Magnetic Resonance, Series A*, 1995, **113**, 210-222.
2. S. Akoka, L. Barantin and M. Trierweiler, *Analytical Chemistry*, 1999, **71**, 2554-2557.
3. N. Michel and S. Akoka, *Journal of Magnetic Resonance*, 2004, **168**, 118-123.
4. S. Heikkinen, M. M. Toikka, P. T. Karhunen and I. A. Kilpeläinen, *Journal of the American Chemical Society*, 2003, **125**, 4362-4367.
5. M. Deschamps, F. Fayon, J. Hiet, G. Ferru, M. Derieppe, N. Pellerin and D. Massiot, *Physical Chemistry Chemical Physics*, 2008, **10**, 1298-1303.
6. N. Hertkorn, C. Ruecker, M. Meringer, R. Gugisch, M. Frommberger, E. M. Perdue, M. Witt and P. Schmitt-Kopplin, *Anal Bioanal Chem*, 2007, **389**, 1311-1327.

7. V. Martin-Jézéquel, M. Hildebrand and M. A. Brzezinski, *Journal of Phycology*, 2000, **36**, 821-840.
8. A. M. Calafat, L.-Y. Wong, Z. Kuklenyik, J. A. Reidy and L. L. Needham, *Environmental Health Perspectives*, 2007, **115**, 1596-1602.
9. M. Houde, A. O. De Silva, D. C. G. Muir and R. J. Letcher, *Environmental Science & Technology*, 2011, **45**, 7962-7973.
10. J. C. D'Eon, A. J. Simpson, R. Kumar, A. J. Baer and S. A. Mabury, *Environmental Toxicology and Chemistry*, 2010, **29**, 1678-1688.
11. A. J. Simpson, D. J. McNally and M. J. Simpson, *Progress in Nuclear Magnetic Resonance Spectroscopy*, 2011, **58**, 97-175.
12. D. S. Gorman and R. P. Levine, *Proceedings of the National Academy of Sciences* 1965, **54**, 1665-1669.
13. R. Soong, E. Nagato, A. Sutrisno, B. Fortier-McGill, M. Akhter, S. Schmidt, H. Heumann and A. J. Simpson, *Magnetic Resonance in Chemistry*, 2015, **53**, 774-779.
14. H. Hummel, C. Amiard-Triquet, G. Bachelet, M. Desprez, J. Marchand, B. Sylvand, J. C. Amiard, H. Rybarczyk, R. H. Bogaards, J. Sinke and L. de Wolf, *Science of The Total Environment*, 1996, **188**, 233-241.
15. G. Nestor, J. Bankefors, C. Schlechtriem, E. Brännäs, J. Pickova and C. Sandström, *Journal of Agricultural and Food Chemistry*, 2010, **58**, 10799-10803.
16. P. Weers and R. Gulati, *Freshwater Biology*, 1997, **38**, 721-729.
17. D. Courtier-Murias, H. Farooq, H. Masoom, A. Botana, R. Soong, J. G. Longstaffe, M. J. Simpson, W. E. Maas, M. Fey, B. Andrew, J. Struppe, H. Hutchins, S. Krishnamurthy, R. Kumar, M. Monette, H. J. Stronks, A. Hume and A. J. Simpson, *Journal of Magnetic Resonance*, 2012, **217**, 61-76.
18. A. Bax, A. F. Mehlkopf and J. Smidt, *Journal of Magnetic Resonance (1969)*, 1980, **40**, 213-219.
